# Low risk of social harms in an HIV assisted partner services programme in Cameroon

**DOI:** 10.1002/jia2.25308

**Published:** 2019-07-19

**Authors:** Beatrice M Wamuti, Thomas Welty, Winifred Nambu, Francois T Chimoun, Ray Shields, Matthew R Golden, Carey Farquhar, Pius T Muffih

**Affiliations:** ^1^ Department of Global Health University of Washington Seattle WA USA; ^2^ AIDS Care and Prevention Program Cameroon Baptist Convention Health Services Bamenda Cameroon; ^3^ Department of Epidemiology University of Washington Seattle WA USA; ^4^ Department of Medicine University of Washington Seattle WA USA; ^5^ Public Health Seattle & King County HIV/STD Program Seattle WA USA

**Keywords:** assisted partner services, HIV case finding, Cameroon, testing, violence, social harms

HIV transmission through heterosexual sex and sex between men accounts for majority of new HIV infections 1. Notifying and offering testing services to partners of people living with HIV can be an effective way of reaching people at higher risk of HIV and linking them to care. Assisted partner notification services (aPNS) has been a routine part of HIV care programmes in US and Europe to increase HIV case finding, and has been implemented at a programmatic level in SSA in Cameroon. Increasingly, it is being promoted through the US President's Emergency Plan for AIDS Relief (PEPFAR) as a strategy for sexual partner tracing, notification and HIV testing 2, 3, 4, 5, 6.

## Background of the Cameroon aPNS programme

Cameroon Baptist Convention Health Services (CBCHS) pioneered an aPNS programme in August 2007 5, and through 2015 has provided aPNS to almost 40,000 individuals (approximately 18,000 IPs and 21,000 sexual partners). Options for aPNS include provider initiated, contract or passive referral 7, 8. However, CBCHS largely utilizes the provider‐referral aPNS approach where a health advisor (HA) directly contacts sexual partners without a waiting period, and without disclosing the identity of the IP. Some SSA countries have implemented aPNS in programmatic and research settings, but none has presented data on the CBCHS scale 9, 10, 11. The World Health Organization (WHO) developed aPNS guidelines, anticipating that more low and middle countries will implement it 8.

A main concern expressed by communities where aPNS is being considered has been the risk of social harms including intimate partner violence (IPV), which refers to any form of physical, sexual, emotional or psychological harm by a current or former partner or spouse 12. A subset of 976 IPs receiving aPNS were interviewed about social harms (2014‐2016). In the one month following aPNS 61 (6.3%) reported relationship dissolution, 15 (1.5%) lost financial support and 11 (1.1%) reported physical harm, with none requiring hospitalization 13. Of the 11 who reported physical harm, three attributed the harms to aPNS. HAs provided IPV counselling and support to all participants.

To the best of our knowledge, the CBCHS aPNS programme is the longest running programme of its kind in SSA. The safety data presented include prospective follow‐up on a large number of aPNS recipients in a mature programme. Our findings suggest that despite the high prevalence of reported IPV in West Africa (41.8%; 95% confidence interval: 32.9%, 50.6%) 14, the CBCHS aPNS programme experienced low IPV. While this is reassuring, aPNS programmes should continue to screen IPs for IPV and proceed cautiously when such history is elicited. However, other potentially adverse outcomes were also reported – 6% reported relationship dissolutions, some of which may have been related to the aPN. Although relationship dissolution may not always be a negative outcome for couples in ill‐matched or unhappy relationships, greater understanding of this and the longer‐term consequences would be helpful to guide counselling for aPN.

Our findings are consistent with prior studies which show aPNS is infrequently associated with social harms 5. Although physical IPV has not been reported at increased rates after aPNS 5, 9, there were concerns that aPNS could lead to social harms in a large programme. Among 240 persons participating in two randomized aPNS trials in Malawi, three experienced partnership dissolution and none experienced IPV 11, 15. A larger cluster randomized trial found that 33 (2.9%) of 1119 persons receiving aPNS in Kenya experienced IPV after their partners were notified and tested, with none related to aPNS 9. Finally, a small aPNS pilot study in Mozambique observed three partnership dissolution cases among 206 aPNS recipients with no episodes of IPV 10. These studies all suggest that IPV and other social harms from aPNS are rare. However, they may not have provided a real world, programmatic assessment of aPNS safety.

## Strategies to minimize the risk of social harms

Based on CBCHS experiences, countries/programmes contemplating aPNS implementation can consider the following strategies to minimize the risk of social harms. First, the use of HAs, that is, individuals trained on provision of HTS and aPNS. CBCHS implements aPNS by interviewing IPs and their sexual partners using HAs, who include nurses, peer educators, laboratory technicians, chaplains and support group coordinators. HAs obtain verbal consent to interview IPs about their sexual partners, agree on notification approach, offer HTS to their sexual partners as described by WHO 8 and support IPs and their sexual partners as need arises. HAs advise IPs that aPNS is voluntary, and declining aPNS does not affect their care.

Second, rigorous IPV screening and management. HAs are trained on a specific IPV screening protocol to identify, counsel and refer IPs with recent history of social harm or IPV (within three months), or who fear for IPV after aPNS. IPs with high risk of IPV are referred for gender‐based violence counselling services, and have the notification approach either customized to ensure their safety – for example, changed from provider to contract referral – or deferred. Those with low risk of IPV are cautioned on potential social harms, for example, loss of privacy in the unlikely case their partners inadvertently discover their identity. IPs are encouraged to notify the HA by phone in case of social harms after aPNS in order to receive individual/couple counselling and support. HAs follow‐up on IPs who sustain IPV to ensure participant safety, and offer counselling and referral services.

Third, engaging the IP during selection and conduct of preferred partner notification strategy, while maintaining strict confidentiality. All HA training concludes with HAs taking a formal verbal and written commitment to maintain confidentiality. HAs notify sexual partners in person or by phone that they were exposed to HIV, conduct pre‐test counselling and offer HIV testing or refer the sexual partner to a clinic for HIV testing. HAs follow‐up to confirm that the sexual partner tested for HIV and record the test results. The HAs also refer all IPs and HIV‐positive sexual partners for HIV care and treatment services, link HIV‐positive pregnant IPs or sexual partners to PMTCT services and educate all clients on HIV prevention and risk reduction.

## Conclusions

aPNS, although infrequently associated with IPV, augments HIV testing, diagnosis and linkage to care for IPs and their sexual partners.
If aPNS is implemented in a voluntary and informed way, offering different approaches and support for those who fear IPV, the risk of social harms is minimized.
Countries should consider implementing aPNS to reach undiagnosed HIV‐positive individuals and to link them to care by training HAs to implement aPNS and to manage social harms.

## Competing interests

MRG has received research support from GSK and Hologic.

## Authors’ contributions

PT, WN and TW supervised the implementation of the CBCHS programme and participated in designing this secondary analysis along with MG and CF. PT, WN and TW reviewed safety of programme participants. TW, WN, MRG, CF, RS and BW were responsible for the statistical design of the analysis. BW and WN analysed the data. BW and TW wrote the initial draft of the paper. All authors critically revised, read and approved the final manuscript.
